# Symptoms Reported by Head and Neck Cancer Patients during Radiotherapy and Association with Mucosal Ulceration Site and Size: An Observational Study

**DOI:** 10.1371/journal.pone.0129001

**Published:** 2015-06-10

**Authors:** Anne Margrete Gussgard, Asbjorn Jokstad, Robert Wood, Andrew J. Hope, Howard Tenenbaum

**Affiliations:** 1 Princess Margaret Cancer Centre, Toronto, Ontario, Canada; 2 Faculty of Dentistry, University of Toronto, Toronto, Ontario, Canada; 3 Department of Dentistry, Mount Sinai Hospital, Toronto, Ontario, Canada; 4 Division of Periodontology, Tel Aviv University, Tel Aviv, Israel; Second University of Naples, ITALY

## Abstract

**Background:**

Self-reported pain and impairment of oral functions varies markedly and often in spite of extensive oral mucositis (OM). The aim of the current study was to appraise how patient-reported debilitation caused by OM is influenced by the extent and possibly location of the OM lesions.

**Methods:**

Patients with head and neck cancer undergoing radiotherapy were examined before treatment, twice weekly during 6-7 weeks of therapy, and 3-4 weeks after therapy completion. OM signs of 33 participants were evaluated using the Oral Mucositis Assessment Scale (OMAS), while OM symptoms were recorded using Patient-Reported Oral Mucositis Symptom (PROMS)-questionnaires. Changes in OM experience as a function of OM signs was undertaken by comparing the aggregated and individual PROMS scale values at the point of transition of OMAS ulceration scores between 0 to 1, 1 to 2 and 2 to 3, respectively in the nine intra-oral locations designated in the OMAS. ANOVA with pairwise contrasts using the LSD procedure was applied for comparisons of mean changes of PROMS scale values for the participants who experienced an OMAS score of 2 or more during therapy (n=24).

**Results:**

Impairment of eating hard foods was more when the OMAS score for ulceration anywhere in the mouth or in the soft palate changed from 1 to 2, compared to between score 0 and 1 (p=.002 and p=.05) or between score 2 and 3 (p=.001 and p=.02). Mouth pain increased more upon transition of OMAS score anywhere in the mouth from 1 to 2 compared to 0 to 1 (p=.05).

**Conclusion:**

The relationship between patient-reported impairment of oral function and pain caused by OM ulceration is not linear, but rather curvilinear. Our findings should prompt investigators of future interventional trials to consider using a less severe outcome than maximum OM scores as the primary study outcome.

## Introduction

Head and neck (H&N) cancer patients often experience mouth pain. The mouth pain may be due to the spread of the original tumour, due to surgery, or by the development of oral mucositis (OM) as a toxic side effect of radiotherapy or chemotherapy [1]. Patients with cancer have numerous questions about pain and whether and how pain can be managed during their treatment [2,3]. The patient’s experience of pain is modulated by intrinsic dimensions such as adaptive coping style, co-morbidity, subjective need of analgesics, psychological duress or depression, e.g., due to fear of permanent disfigurement and likely, previous experiences of severe pain [4]. A range of extraneous factors can also influence the patient experience of pain, ranging from the positive effects of emotional support from professionals, family or social network [5], to the negative effects of social isolation.

Mouth pain associated with the H&N cancer therapy is a significant contribution to emotional duress and often leads to lower food intake potentially resulting in undernourishment and weight loss [6]. The level of suffering caused by the mouth pain can extend to such level that the patient may request a lowering of the intensity of the radiotherapy or even renounce further cancer therapy. Many regard the extent of visual manifestation of OM as a proxy for the degree of mouth pain, although perhaps somewhat surprisingly, the scientific evidence for this presumption does not seem entirely justified (Table 1), [7–13]. Patients with extensive OM often report significant mouth pain, despite use of analgesic medication [14]. The OM-derived pain appears to be associated with neurobiological etiological mechanisms [15,16], although the exact details remain unknown. The type of pain in H&N cancer patients appears to be predominantly nociceptive or mixed nociceptive and neuropathic pain [17]. Clarifying the principal pain in H&N cancer patients through detailed description of how the patients report the history and presence of neurological dysfunction may provide indications that can have implications for clinical practice and research. Moreover, it is essential to understand how the H&N cancer patient experiences his or her mouth pain during cancer therapy, to institute possible interventions that could decrease their levels of suffering. Reducing or at least explaining to the patient how pain will affect their daily activities may lower patient anxiety, bolster the compliance with cancer therapy [18] and likely make it easier for the patient to endure comprehensive intraoral examinations. Since the OM-associated pain contributes to total mouth pain (i.e. in addition, say, to pain associated with surgical resection of the tumour) it is imperative to identify interventions that may prevent or reduce the development of OM.

**Table 1:** Prevalence of severe oral mucositis during radiotherapy of H&N cancer patients and patient-reported mouth pain.

Lead author	N	UICC-cancer stage*	Radiotherapy	Prevalence (OM Grade)	Clinical OM assessment	Pain reporting during therapy
Nutting CM et al. 2011 [7]	94	T1-4/N0-3/M0	60–65Gy CRT vs. IMRT	61% vs. 63% (Gr. 3+4)	NCICTCAE v3	Likert scale; Grade 0–4
Murphy BA et al. 2009 [8]	75	T1-4	n.r.Gy CRT vs. IMRT +/- chemo	95% vs. 66% (Gr. 3+4)	OMWQ-HN	Likert scale; Grade 0–4 (MTS)
Palazzi M et al. 2008 [9]	149	T1-4	66–74 Gy IMRT/CRT/3dCRT	28% (Gr. 3+4)	NCICTCAE-v3	Likert scale; Grade 0–4
Elting LS et al. 2007 [10]	204	T1-4/N0-3	64–70Gy IMRT/CRT +/- chemo	66% (Gr. 3+4)	NCI-CTCv.2	Likert scale; Grade 0–4
Urbano TG et al. 2007 [11]	30	T2-4/N0-3	63Gy vs. 67Gy (All + Chemo)	67% vs. 40% (Gr. 3)	NCI-CTCv.2	Pain Y/N
Wendt TG et al.2006 [12]	38	T2-4/N0-3	60–70Gy 3D-cIMRT	11% (Gr. 3)	RTOG	Likert scale; Grade 0–6
Bentzen SM et al. 2001 [13]	918	T2-4/N1-3	54Gy CHART vs. 66Gy CRT	60% vs. 44% (Gr. 4)	EMS(Dische-89)	Likert scale; Grade 0–3

* UICC = International Union against Cancer, (http://www.uicc.org/resources/tnm/publications)

Other acronyms used in table 1:

Gr.: Grade

CRT: ChemoRadiotherapy

IMRT: Intensity-modulated radiation therapy

RT: Radiotherapy

CHART: Continuous Hyperfractionated Accelerated Radiotherapy

NCICTCAE: National Cancer Institute Common Terminology Criteria for Adverse Events

OMWQ-HN: Oral Mucositis Weekly Questionnaire-Head and Neck cancer

RTOG: Radiation Therapy Oncology Group

EMS: Elements of Morbidity System

MTS: mouth and throat soreness

**Fig 1:**
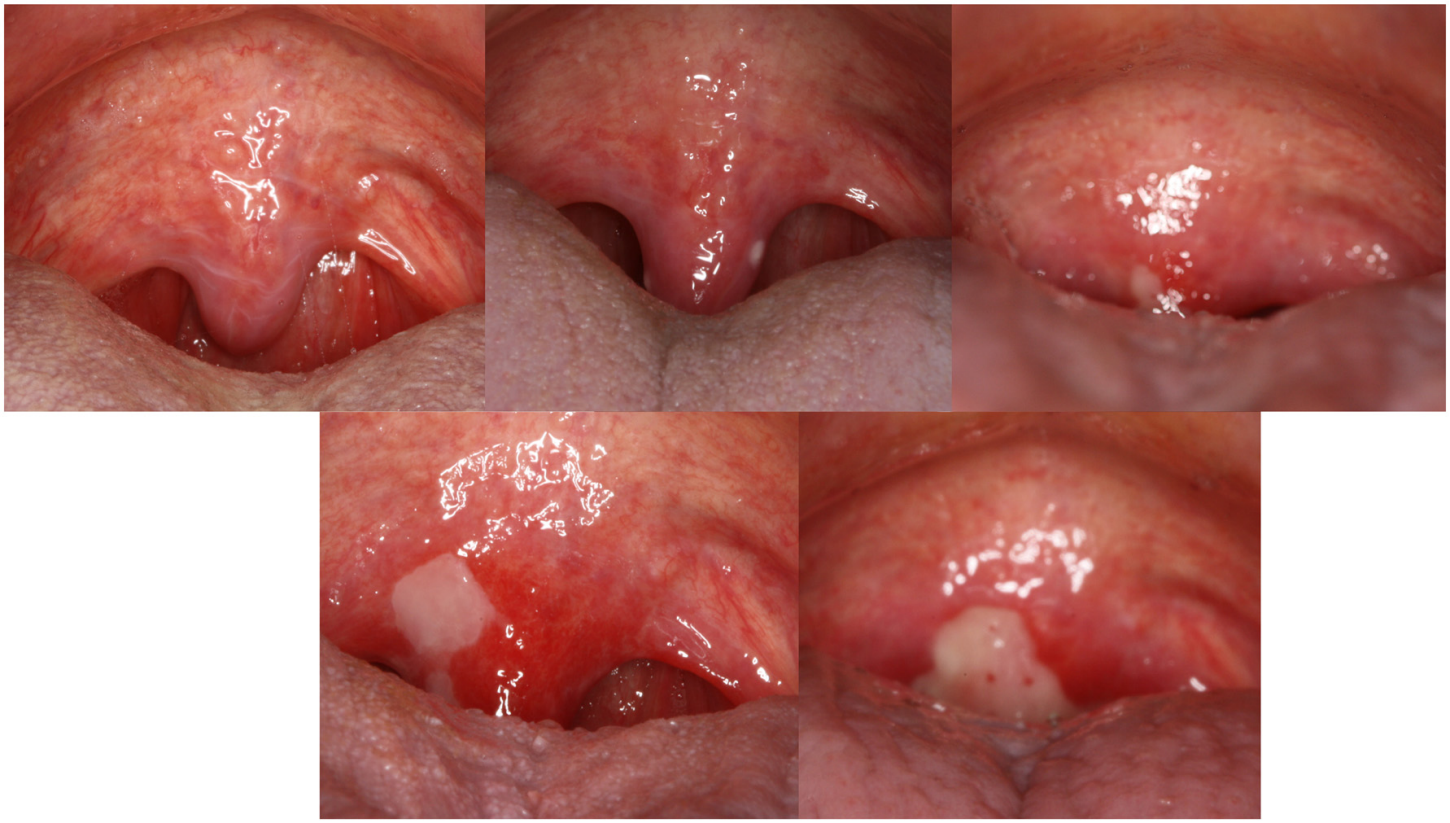
Development of oral mucositis ulceration in the soft palate. In this participant, the first sign of ulceration developed during the 3^rd^ week of radiotherapy on the uvula (upper centre picture). The size of the ulceration increased over the subsequent weeks 4 (upper right picture), 5 (bottom left picture) and 6 (bottom right picture). A common challenge in the clinic examination is that pain and impaired control of pharyngeal and extrinsic tongue muscles caused by the oral mucositis often counteracts a clear visual examination of the back of the mouth and throat.

The current investigator group recently appraised the merits of adopting a new patient-reported oral mucositis experience instrument named PROMS (Patient-Reported Oral Mucositis Symptom) [19] in a cohort of H&N cancer patients [20]. The main purpose of this observational study was to elucidate whether the OM that affected the study participants during the course of their radiotherapy correlated with signs of OM. The investigators undertook detailed intra-oral examinations that included clinical scoring of OM according to the Oral Mucositis Assessment Scale (OMAS) protocol [21] twice per week while the participants underwent radiotherapy (Fig 1). Upon applying Spearman rank correlation tests in repeated-measures mixed linear models between the PROMS scale values and three different clinician-based scores at various time points while the patients underwent radiotherapy it was apparent that the patient experiences of OM correlated well with the scoring tools on a group basis [20]. However an intriguing observation in the investigation was that some participants reported hardly any mouth pain, in spite of visual manifestation of large and often confluent areas of ulcerations of the intraoral mucosa, and vice versa. These observations led the current investigator team to explore how the participants’ self-reported mouth pain was associated with the intraoral location and extent of OM lesions. The working hypothesis was that the advent of patient-reported debilitation due to OM was influenced by the extent and possibly location of the OM lesions.

## Materials and Methods

The materials and methods have been described in detail elsewhere [20], and the data presented in this paper are based on secondary analyses of the main study.

### Main study

In brief, a prospective single cohort study was undertaken at the Princess Margaret Hospital/Ontario Cancer Institute (PMH). The objective was to appraise the merits of supplementing clinical assessments of OM with the PROMS instrument amongst H&N cancer patients undergoing radiotherapy with or without concurrent chemotherapy. Study approval was obtained from the Research Ethics Boards of the Toronto University Health Network in 2009 (ref. #09-0231-CE) and written informed consent was obtained from all study participants. Twenty participants were required to obtain 80% power of the study, based on estimation of 90% correlation between self-reported and observed data. In expectation of a high participant dropout, the investigators recruited more participants than strictly required.

Eligible participants were identified by being 18 years of age or greater and diagnosed with carcinoma in the head and neck region and a minimum Karnofsky score performance status of 60%. The 60% lower threshold was chosen for logistical as well as ethical reasons, as patients below this score require considerable assistance that would introduce a disproportionate burden for the participant and the hospital support staff. All participants were scheduled to receive radiotherapy for their H&N cancer with a minimum prescription radiation dose of 54Gy, with or without concurrent chemotherapy.

Fifty consenting participants underwent an oral examination at baseline prior to the commencement of cancer therapy. Seven participants did not complete the cancer therapy and three received less than the 54 Gray radiation dose while 7 discontinued their participation in the current study, primarily due to fatigue. The remaining 33 participants were examined clinically twice-weekly over their course of seven (n = 25), six (n = 7) and four (n = 1) weeks of radiotherapy, and then one more time four to six weeks after the completion of the cancer therapy.

The visual manifestation of OM was appraised clinically in accordance with the OMAS-instrument as described by its developers [21]. An assessment of ulceration or erythema was made in nine different intra-oral locations (upper lip, lower lip, right and left cheek, right and left ventrolateral tongue, floor of mouth, soft palate and hard palate). Scores were assigned values of 0, or 1 to 3 according to the extent of mucositis. For ulceration, scores 1 and 2 denote an ulcerated area, respectively of less than, and more than 1 cm^2^, and a score of 3 denotes an area of more than 3 cm^2^. The clinical examiners were calibrated prior to the study initiation by using photographs developed for such purposes, and laminated photographs were used during the study to avoid drifting of the intra-rater assessments.

At each clinical examination, the participants completed a PROMS questionnaire [18] to appraise how OM affected common daily oral functions. The PROMS scale consists of 10 questions that are answered on a visual analog scale (VAS), by setting a mark on each horizontal line measuring 100 mm. Two questions focused on mouth pain and change in taste ranging from none to worst possible and complete change in taste, respectively. The other 8 questions focused on how much their mouth sores impaired different oral functions on the day of the clinical examination. Memory of pain or other dysfunction was not requested, on grounds of being deemed unreliable. Impaired oral functions included difficulty with speaking, swallowing, drinking or eating hard or soft foods as well as restriction of eating, drinking or speech. The participants were also solicited about any intake of analgesic medication or necessary in-hospital stay, with or without required nutritional support through tube feeding in-between the clinical examinations. The participants were consistently asked at every visit whether they felt a need to discuss with the investigator any issues regarding oral dysfunction, including mouth pain and pain management during the course of their radiotherapy.

### Secondary analyses

The secondary analyses aimed to determine whether there was an association between oral mucositis symptoms and any specific extent or location(s) of visually manifest OM. In this perspective, the changes of the aggregated and individual PROMS scale values were measured when changes were identified between OMAS score 0 to 1, 1 to 2 and 2 to 3, respectively in any of the nine intra-oral locations designated in the OMAS [21]. Prior to being subjected to parametric or non-parametric statistical tests for comparative purposes, the mean changes of PROMS scale values were checked for normal distribution and any need for log-transformation corrections. ANOVA with pairwise contrasts using the LSD procedure were applied for comparisons of mean changes of PROMS scale values upon transition between the three levels of OMAS scores 0 to 1, 1 to 2 and 2 to 3, with the hypothesis that the PROMS changes are equal to each other (IBM SPSS ver. 22, IBM Corporation, Somers, NY).

## Results

### Demographics

Twenty-four of the 33 participants provided data that enabled the appraisal of change of the PROMS scale value changes as a function of OMAS score changes. Six participants did not experience oral mucositis beyond an OMAS score of “1”. Two participants had missing clinical scores or self-reported PROMS scale data, and one participant received radiotherapy over four weeks only. Of the 24 participants, half received concurrent chemotherapy (n = 12, 50%). The cohort was predominantly Caucasian (n = 20, 83%), and consisted of 19 males (79%). The average age was 60 years (range 38–78 years). The proportion of never-smokers was 25% (n = 6), ex-smokers 50% (n = 12) and smokers 25% (n = 6). The participants were diagnosed with either carcinoma of the oral cavity or oropharynx (n = 14, 54%), or in the salivary glands, nasopharynx, maxillary sinus or primary site unknown.

Twenty-two of the 24 reported that they self-administrated analgesic medication more or less constantly during the course of the cancer therapy. The type of medication varied, but often included opioids. Despite this medication, the participants reported consistently on the PROMS VAS-forms that they experienced mouth pain throughout the entire period of cancer therapy.

### OMAS and PROMS measurements

**Fig 2:**
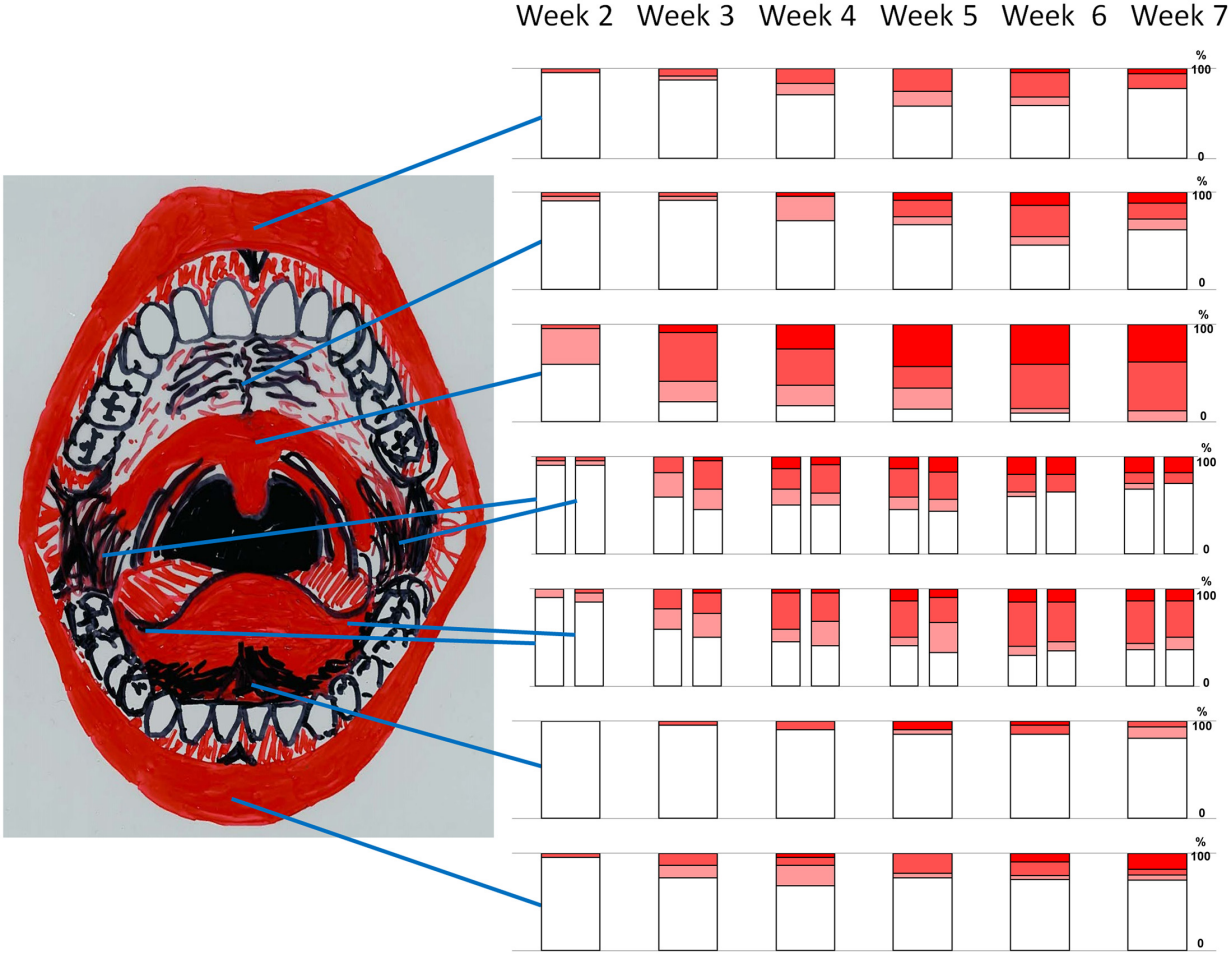
Development of oral mucositis ulceration from week 2 of the 7 weeks cancer therapy period. Nine locations shown, in accordance with the OMAS scoring system (Sonis et al. 1999). From top is shown the ulceration status of the: upper lip, hard palate, soft palate, right and left cheek, right and left ventrolateral tongue, floor of mouth and lower lip. Percentage of OMAS score 0 (no mucositis) = white; Percentages of OMAS scores 1,2 and 3 = increasing shading.

The OMAS scores in this study cohort increased progressively towards the end of the cancer therapy period and for some patients ulcerations were visually manifest as early as the 2^nd^ week of radiotherapy. The predominant intra-oral locations of the ulcerations were the soft palate, cheeks and right and left ventral and lateral tongue. The upper and lower lips were involved less frequently, and OM in the floor of the mouth was reported only to a small extent (Fig 2).

**Fig 3:**
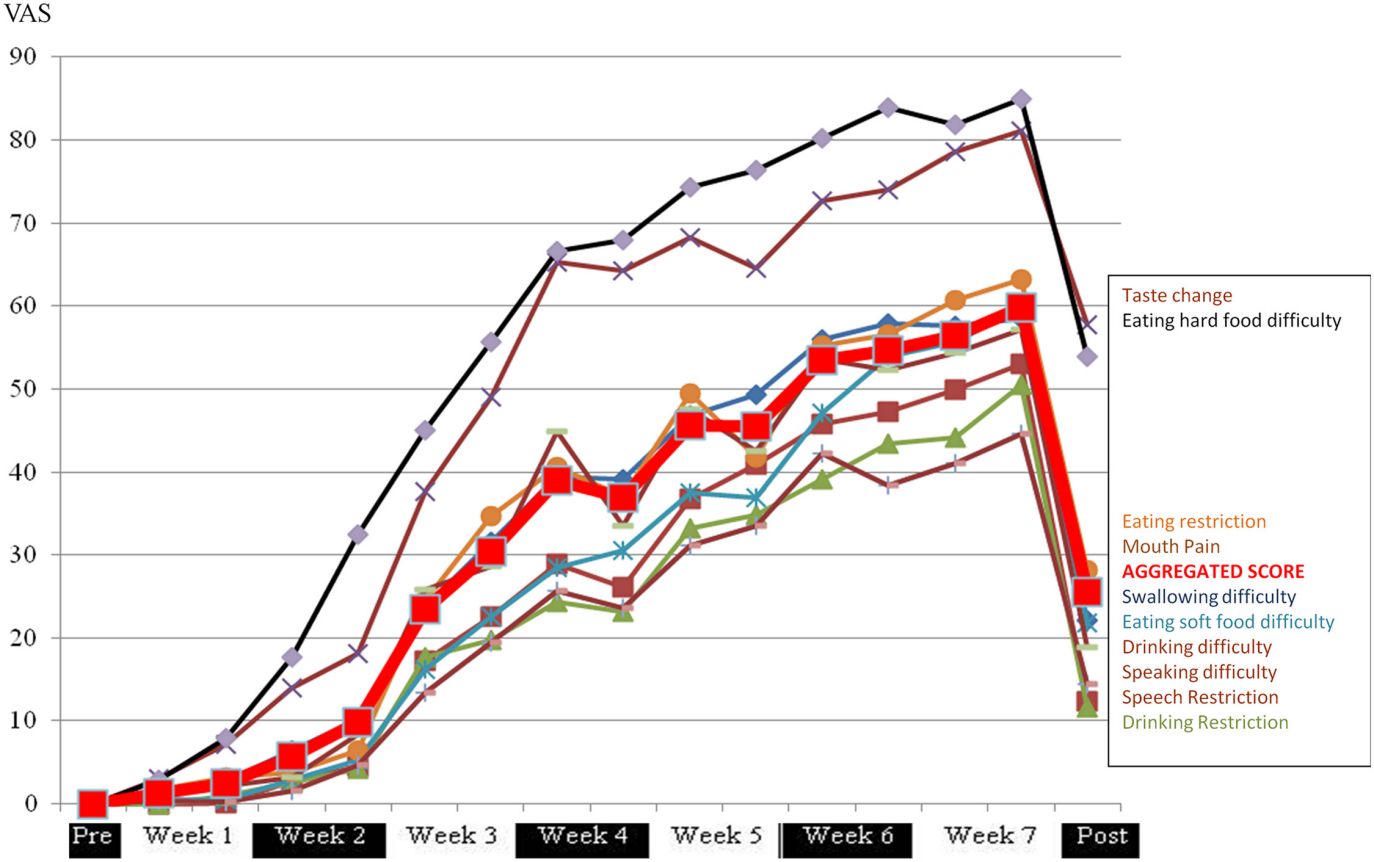
Patient-reported PROMS scale VAS-values experienced over the full course of the 7 weeks cancer therapy period. Left side indicate the mean PROMS scale VAS-values at baseline “Pre” before commencing therapy. Right side show the mean PROMS scale VAS-values at the post-therapy examination 4–6 weeks after the completed cancer therapy (“Post”). The mean aggregated PROMS scale average is emphasized in red, while the 10 separate components of the PROMS instrument (listed to the right) are shown in different colors. Higher VAS-values denote more impairment of oral functions (max VAS = 100).

The VAS values for all the ten components of PROMS, as well as the aggregated average, increased gradually during the cancer treatment period. “Change of Taste” and “Difficulty eating hard foods”, were considerably more affected by OM than the other 8 components of the PROMS (Fig 3).

### PROMS and OMAS association

The changes in extent and severity of intra-oral visually manifest erythema seemed to have little influence on the change of patient-reported PROMS scale values amongst the 24 participants (data not shown). The changes in visually manifest ulceration on the other hand, appeared to closely relate to changes in the PROMS scale values.

**Fig 4:**
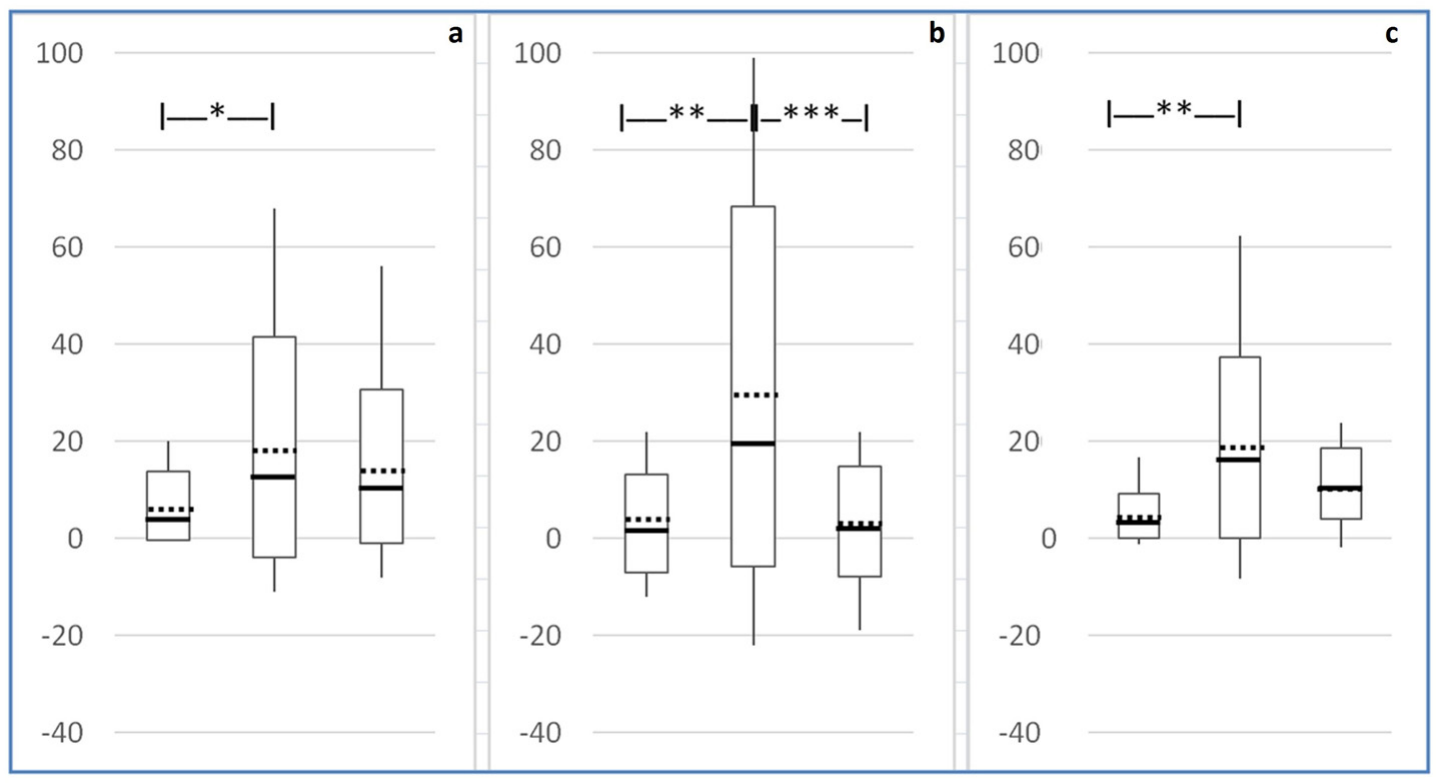
Change of patient-reported PROMS scale VAS-values upon transitions between OMAS scores 0 to 1, 1 to 2 and 2 to 3 anywhere in the mouth. The three boxplots within each graph show the dispersion of changes in VAS-values of mouth pain (a, left), difficulties eating hard food (b, centre) and aggregated PROMS (c, right) upon the transitions (maximum change = VAS value 100). The interrupted horizontal lines in the box centers represent the mean changes, with the upper and lower box edges indicating the SD. The horizontal full lines represent the median, and the whiskers represent the maximum and minimum changes of VAS-values. Horizontal bars above box-plots indicate statistical significant difference of PROMS change (ANOVA with LSD pairwise contrasts (* = P< 0.05, ** = p<0.01, *** = p<0.001)).

Upon transition between OMAS ulceration scores 0 to 1, 1 to 2 and 2 to 3 anywhere in the mouth, the PROMS scale values changed more between the shift from scores 1 to 2, than between the shift from scores 0 to 1 or between the shift from scores 2 to 3 (p = 0.009). Patient reported difficulties with eating hard food due to mouth sores anywhere in the mouth changed also more between the shift from scores 1 to 2, than between the shift from scores 0 to 1 or between the shift from scores 2 to 3 (p = 0.001). In general, upon transition of ulcerations from scores 1 to 2 anywhere in the mouth, there was a tendency that the relative increase of mouth pain, and eating hard foods and the aggregated PROMS scale values appeared to be higher compared to the shift from scores 2 to 3 (Fig 4).

**Fig 5:**
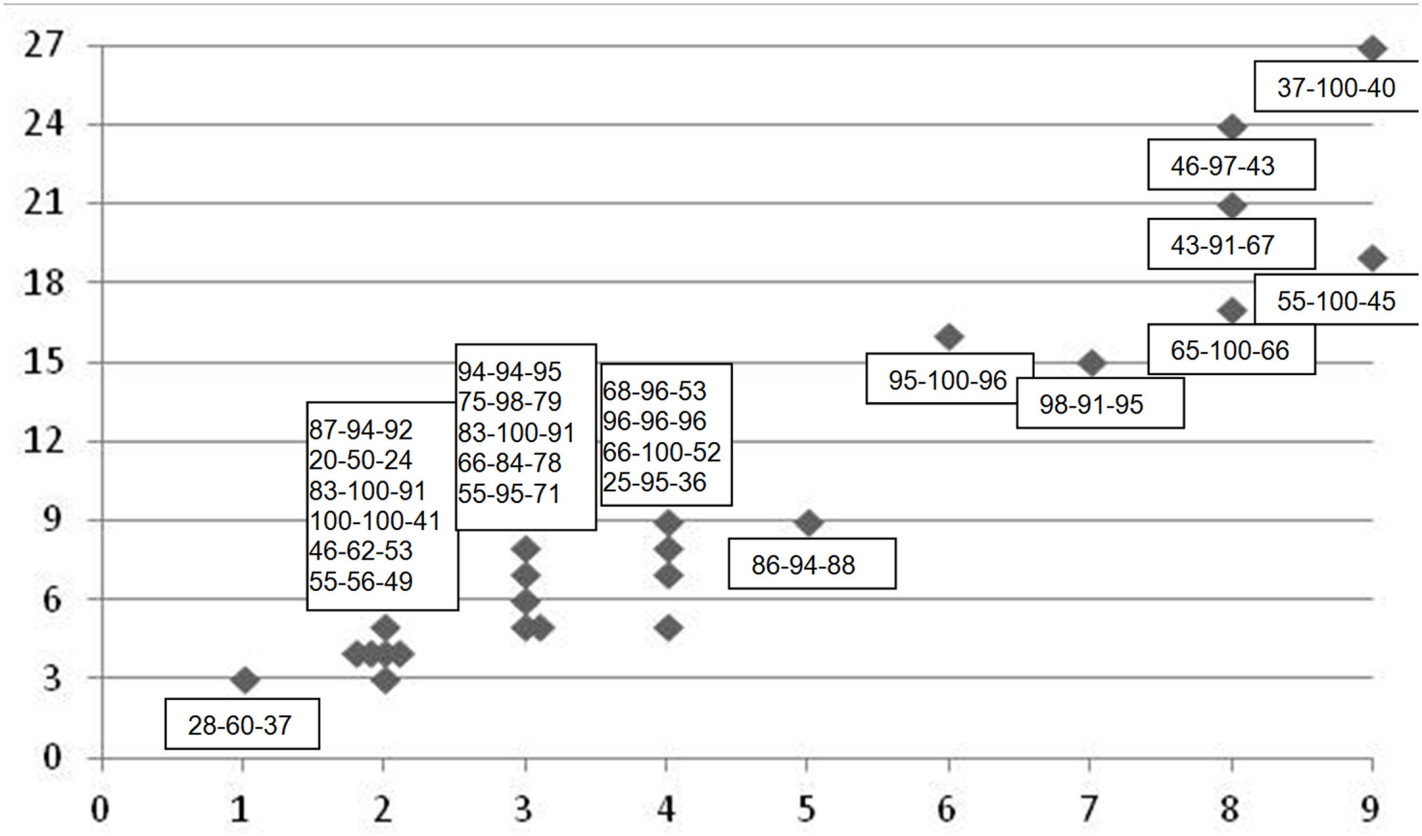
Oral mucositis ulceration score and PROMS scale VAS values recorded on the last radiotherapy session of the 7 weeks cancer therapy period. The horizontal axis shows the observed number of intra-oral sites with ulceration (max = 9). The vertical axis indicates the accumulated OMAS score of the ulcerations (max = 27). The boxes show the individual participants’ PROMS scale VAS values for: Pain—Difficulty eating hard food—Aggregated PROMS average. Higher VAS-values denote more impairment of oral functions (max VAS = 100).

The majority of participants experienced visually manifest ulceration in two to four sites (Fig 5). One participant had OMAS score 3, i.e., more than 3 cm^2^ in one site, while two participants suffered from OM in all 9 intra-oral sites and of these two, one had the maximum OMAS score of 3 in all nine sites (i.e., OMAS score 27, Fig 5). Both reported relatively medium mouth pain and average PROMS scale values (VAS 37–55), but severe (VAS = 100) impairment of eating hard foods.

## Discussion

The study cohort can be characterized as heterogeneous, in terms of participant age, dental status, smoking and alcohol intake, primary tumour location, TNM cancer stage, surgery excision or not, use of supplementary chemotherapy, therapy length and severity of OM. To clarify to what extent these factors individually or in concert affect patient-experienced mouth pain during cancer therapy can only be determined in a far larger study. The logistical, ethical and practical challenges upon conducting studies that necessarily will require multivariate, multilevel statistical analyses of a large sample size to address such issues are likely reasons why these potential associations to a little extent have been elucidated. The size of the current sample is small and was not originally designed to test correlation between size and/or location of oral mucositis ulcerations and patients’ experiences of OM. The risk of potential bias introduced by conducting post-hoc analyses is acknowledged. Still, to the authors’ knowledge, the assumed linear correlation between mouth pain and extent and location of OM has not been addressed before by any investigators. Moreover, while the size of OM might not have the expected impact on pain as ordinarily expected, it could still impair functions that are equally, if not more important to the patient, than pain alone.

The participants in this study did not report mouth pain during their first week of radiation therapy, which is at odds with other studies suggesting that about 50% of H&N cancer patients have pain prior to cancer therapy [22,23]. One possible explanation of the apparent discrepancy may be that PROMS-questionnaire focus on effects of actual mouth sores (i.e. oral mucositis) and the question about mouth pain was also considered within this context [19]. To emphasize this element further, the information that *“the mouth encompasses also lips*, *cheeks*, *tongue*, *gums*, *palate and throat”* was added to the pertinent question on the PROMS questionnaire for clarification, which would likely reduce underreporting of pain. Participants were not requested to describe the qualities or intensity of their pain as modulated by functions. This does not negate that other strategies should also be attempted to hopefully elucidate which factors that aggravate pain in H&N cancer patients [24].

Best practice to deal with oral mucositis and associated mouth pain is unfortunately not obvious, which is reflected by the most updated evidence-based guidelines recently developed by the Multinational Association of Supportive Care in Cancer (MASCC) and The International Society of Oral Oncology (ISOO) [25]. Part of the conundrum is our incomplete understanding of how radiotherapy-induced OM affects the cancer patient. In this context, our findings that patients report relatively more problems upon transitions from small to medium size visually manifest OM rather than between medium to larger confluent ulcerations or between none to minor size OM is of high clinical relevance.

Our current understanding of pain associated with cancer is inadequate and attempts to elucidate the etiopathogenesis is tempered by both patient expectations of pain and symptom reporting, as well as clinician perceived perceptions of effectiveness [26–28]. The patient-reported high intake of analgesics and impression of poor effects noted in the current study corroborates observations made in other clinical studies. Poor analgesic control may indicate that the pain mechanisms involved during the radiotherapy of H&N cancer patients may have a neuropathic rather than a strictly nociceptive component. Neuropathic cancer pain is associated with a negative impact on activities of daily living and greater requirements for analgesics than nociceptive cancer pain [29].

In the current investigation, the participants had a Karnofsky performance status of minimum 60%. To what extent this affects the external validity of the results to more disabled patients is uncertain. The current consensus is that pain symptoms and associated psychological distress does not appear to be influenced by Karnofsky scores [30,31]. Most of the patients developed mucosal ulcerations on their soft palate and/or tongue although the variability was great. The combination of large variability and small study sample cautions against making any strong inferences, but it appears that the location of a lesion could be more important insofar as oral functions are concerned than merely size of the lesion. Ulceration in the soft palate caused a major increase in problems eating hard food as well as reported pain, when the OMAS score for ulceration changed from score 1 to 2 (Fig 4). This change could be due to increased swallowing sensitivity resulting from soft palate ulceration. Patients may be able to more or less ignore an OM ulceration that is less than 1cm^2^ (OMAS score 1) in this location, but that when exceeding 1cm^2^, they certainly are affected and their PROMS scale values increase.

When there is extensive OM there are likely several ongoing transitions between OMAS scores 0 to 1, or 1 to 2, alternatively from 2 to 3 simultaneously in several areas intra-orally. The sum of these mouth sores influence the patient when he or she reports the level of suffering by marking on the VAS scale in the PROMS questionnaire. The soft palate was more affected by OM than the other locations during the early stages of the radiotherapy. It is therefore important to realize that pain experienced upon OMAS transition from score 0 to 1 is less in the soft palate compared to when the transition occurs in other intra-oral sites.

An element that was not measured in the current study, but needs to be considered is whether the depth of the OM lesions is associated with the extent of pain or functional debilitation. Optical Coherence Tomography is a technique that may be used for detecting OM before visual manifestation, but with the current state of the technology contrasts become blurred when the OM develops [32].

The association between the individuals’ PROMS-scale values with the OMAS scores (Fig 5) did not demonstrate any clear patterns. The small study sample precludes the possibility to draw too many conclusions in this regard. Severe impairment of oral functions was reported by some participants with ulcerations limited to two or three sites. Alternatively, six of the worst affected in terms of amount of intra-oral ulcerations reported only modest mouth pain, as defined by a VAS-values between 37 mm and 65 mm, while some of the individuals with ulcerations limited to two or three sites reported VAS values above the 80’s (Fig 5). The size of the ulceration itself is important, but Fig 5 shows that an increased number of ulcerations may not necessarily contribute to more pain than having just one ulceration. The observation that a single ulceration above a certain size may cause major discomfort for a patient is consistent with the statement made in the original OMAS-study paper that: “…*worst site and extent of severe mucositis appeared to be more responsive to change [in mucosal health] than mean mucositis score”* [21].

Clinical studies that select as primary outcome the most severe visually manifest OM scores select a clinically relevant outcome. However, measurements of a less severe level of visually manifest OM appear to be more patient relevant. The current study showing that patients report pain and significant impairment of oral functions even when scores are lower than e.g. OMAS score 3 or WHO (World Health Organization) score 3 or NCI-CTCAE score 3 is obviously clinically relevant.

The current observations that the major change in PROMS scale values occurs upon transition from small to medium, rather than from medium to large visually manifest ulcerations corroborate observations findings reported by Elting et al. [10]. Although these investigators worded that *“oral pain scores peaked earlier than the maximum grade of OM”* the essential interpretation is that the size of OM ulceration above a certain level does not necessarily lead to more pain.

## Conclusion

The development of one or more ulcerations with surface areas of less than approximately 1 cm^2^ does not impair oral functions much, as measured with the PROMS questionnaire. However, an increase of any ulceration surface area to more than 1 cm^2^ cause a relatively large change of reported impairment and mouth pain, which was larger than the relative change upon transition of any ulceration area from less than to more than 3 cm^2^. Hence, the relationship between patient-reported impairment of oral function and mouth pain caused by OM ulceration is not linear, but rather more curvilinear.

Clinical trials that select the maximum visual manifest OM score as primary outcome, such as OMAS score 3, NCI scores 3 and 4, WHO score 3 to assess intervention efficacy, select a clinically relevant outcome. However, the observations made in the current study would suggest that a less severe primary outcome may be more patient-relevant. Further and larger clinical studies are needed to appraise the association between severity of OM and patient-experienced pain and dysfunction.

## Supporting Information

S1 File Raw Data matrix. This supporting information file is a Microsoft Excel file containing all raw data. Three separate sheets contain data from the Case Report Forms, OMAS clinical scores and PROMS scores respectively. Column #1 is the case identifier number of the 50 participants. The top row contain the names of the variables. Further details about this data matrix can be addressed to: anne.m.gussgard@uit.no.
(XLSX)
